# Publishing challenges for perfusionists whose first language is not English

**DOI:** 10.1051/ject/2024024

**Published:** 2024-09-20

**Authors:** Raymond K Wong

In preparing the last several issues, I have been gratified that JECT has been able to publish quite a few articles from non-native English speaking countries, especially since our journal became openly accessible worldwide in 2023. While it’s nothing new for JECT to accept international submissions, it has continued to be a challenge to ensure that such manuscripts are handled in a positive, respectful manner, especially when the English grammar and prose are particularly deficient. The easy way out for editors and peer reviewers would simply be to reject such submissions solely based on their inadequate language qualities. Doing so however, would deprive all involved from the benefits of such articles as I will further discuss below. As challenging as it has been for me to work with such manuscripts and their authors, I try not to lose perspective of how much more difficult it can be for some international perfusionists to prepare and submit manuscripts to established English language journals like JECT. It was thus very timely that the Committee on Publication Ethics (COPE) ran a forum this past June entitled “Publishing when English is not your first language” [1]. I will attempt to synopsize and incorporate some of what was presented in this editorial with the goal of raising awareness in the perfusion community (authors, peer reviewers, readers, etc) of the benefits, barriers/challenges and potential solutions for JECT to publish international submissions from non-native English speakers, which includes many from lower and middle income countries and the global south.

The benefits to the worldwide community of being able to publish and read work from non-native English speaking perfusionists are many: hearing innovative, diverse voices can contribute to improving our clinical technique repertoire, skills and knowledge leading to enhanced patient care outcomes. Problems faced by regions speaking one language can lead to solutions being proposed that can be shared with regions speaking another language who face similar issues. A prime example published in this very issue is the problem of unavailability of Plasmalyte-A^TM^ for use in preparing del Nido cardioplegia solutions in some parts of the world, leading two groups in the Middle East to study alternative, modified solutions; the results of which could easily be implemented in other regions of the world sharing this same problem [2, 3]. I am currently sheparding another manuscript by an author from India who presented his work at this year’s AmSECT’s International Conference and won an award for their novel autologous priming technique that can eventually be shared and implemented in both first world and developing countries. Authors from other countries can also occasionally identify and fill gaps in our knowledge and the literature such as the Colombian group in this issue who reviewed cardiac rehabilitation strategies in patients supported on ventricular assist devices [4]. The lack of representation or inequity of non-English speaking voices in the perfusion literature can also be occur with first world countries, such as with authors from Japan. In this issue and in a previous one last year however, we were fortunate to publish work by Takeichi, et al. on their novel innovations to challenges in minimally invasive cardiac surgery [5, 6]. Finally, non-native English-speaking authors benefit when publishing in English journals such as JECT from increased recognition and career satisfaction as well as from being provided with opportunities for professional development.

Non-native English-speaking authors face a variety of barriers and challenges in attempting to publish in English. As I mentioned before, it would be easy for editors and peer reviewers to reject manuscripts with poor English and such occurrences are likely the predominant challenge that non-native English speaking authors face. Their manuscripts are often judged, not based on scientific merit but on presentation. As described by a COPE forum attendee, for non-native English speakers, poorly written manuscripts can result when they think in their native language and translate that into English. However, for them, having to think in English, write in English and revise in English is an extra burden that native English speakers may not appreciate. Even English speakers from some countries, such as former British or American colonies can encounter difficulties since their English usage can be articulated differently, with different expressions and vocabulary usage such that extensive revisions are still required.

So how can journals like JECT help non-native English speakers publish? We will have to work harder – it takes more effort by all involved to produce articles from non-native English speakers. Having diverse representations in our stable of peer reviewers and on our editorial board goes a long way in understanding the challenges and aiding in the form of mentorships, for example. I have and will continue to recruit diverse volunteers to assist with JECT manuscript processing. If you would like to participate, please contact me directly.

During the COPE forum, it was also emphasized that the journal and its peer reviewers should be willing to accept language imperfection, as long as the text is clear and understandable. Originality and clarity should be emphasized over grammar and prose. Moreover, certain expectations that non-native English authors have of themselves could also be discouraged. I have seen manuscripts that provide extensive/discursive introductions that are not necessary for the audience of primarily clinical perfusionists who read JECT. Instead, such authors should focus on describing well, key areas such as the methods and be as concise as possible elsewhere. So both authors and peer reviewers can be provided with additional guidance to produce language-acceptable manuscripts.

The use of cost-effective artificial intelligence (AI) driven language services is one proposed solution in JECT’s instructions for authors but such services have their limitations and any AI use should be declared. More comprehensive services are available, with larger publishers having in-house options which they can sometimes provide waivers for international authors to use. Our sponsoring society, AmSECT as well as other international perfusion professional societies could consider creating sponsorship funds for authors to use such language services.

In full disclosure, I myself was raised trilingual in a developing country, Malaysia, and thus I am cognizant of the opportunities and challenges of publishing when your native language is not English. However, I have had the privilege of expressing my voice and thoughts on these pages and it is certainly my desire and intent for perfusionists worldwide to be able to do so as well. I truly believe that voices from around the world will enrich our perfusion ecosystem.
